# Mycotic aneurysm due to *Salmonella* species: clinical experiences and review of the literature

**DOI:** 10.1590/1414-431X20186864

**Published:** 2018-06-25

**Authors:** Yiqun Guo, Yu Bai, Chunxia Yang, Peng Wang, Li Gu

**Affiliations:** Department of Infectious Diseases and Clinical Microbiology, Beijing Chao-yang Hospital, Capital Medical University, Beijing, China

**Keywords:** Salmonella, Mycotic aneurysms, EVAR

## Abstract

The mortality of patients with mycotic aneurysms is high, especially in East Asia, and infection by *Salmonella* species is the most common. Our study aimed to improve prognosis of adult mycotic aneurysms with early diagnosis and accurate treatment. Four adult patients with mycotic aneurysm caused by *Salmonella* were included and analyzed by single-center retrospective analysis. Cases reported in the literature during the past 10 years were also summarized. The average age of the 4 male patients was 61.25 years, while that of the 53 cases reported in the literature was 65.13 years. Hypertension, diabetes, and atherosclerosis were common complications. Most patients presented fever and experienced pain at the corresponding position of the aneurysm. Laboratory examination found an increased number of white blood cells accompanied by an increase in inflammatory markers. Most aneurysms were found in the abdominal aorta, while the rupture of an aneurysm was the most common complication. The mortality rates were 21.43 and 7.14% after open surgery or endovascular aneurysm repair (EVAR) intervention, respectively. The recurrence rates of infection were 0 and 17.85% for both treatments, respectively. The mortality rate of mycotic aneurysm caused by *Salmonella* infection was high in middle-aged males with hypertension, diabetes, and atherosclerosis. The possibility of a *Salmonella*-infected aneurysm should be considered in these high-risk groups presenting chills, fever, chest, and back pain. Open surgery was superior to EVAR treatment in the clearance of infected foci and the reduction of postoperative recurrence. The recurrence of postoperative infection can be prevented by intravenous antibiotic therapy for 6 weeks post-surgery.

## Introduction

Mycotic aneurysm is rare; however, the disease is severe and develops rapidly. The incidence of rupture is higher than that of the arteriosclerotic aneurysm with a high rate of mortality. Early diagnosis and adequate treatment (active surgical treatment and antibiotic treatment with adequate dosage and duration) are significant for the improvement of survival. In Western countries, approximately 0.5–1.3% of the aneurysms are of bacterial origin, while in Asia, especially East Asia, the proportion of a bacterial aneurysm is significantly higher. Previous studies reported that about 13.3% of the aneurysms have bacterial origin (1,2), among which Salmonella infection is common, second only to *Staphylococcus aureus* (3).

The present study collected and retrospectively analyzed the 4 cases of patients with adult mycotic aneurysm caused by *Salmonella* admitted at the Beijing Chao-yang Hospital affiliated to Capital Medical University since 2007. In addition, the cases reported in the relevant literature from China and worldwide since January 2007 were reviewed and analyzed. The clinical characteristics and treatments of adult mycotic aneurysms caused by *Salmonella* were summarized.

## Material and Methods

### Study design

This study was a single-center retrospective case analysis of the patients who were treated at the Beijing Chao-yang Hospital affiliated to Capital Medical University. The drug selection and surgical approaches by physicians according to the patient's condition were examined. In addition, the literature from the previous 10 years was summarized.

Given the retrospective nature of the study, written consent was not obtained. However, we had oral consent from all participants by telephone contact, and patient records were anonymized and de-identified prior to analysis. Then, related data were extracted from the hospital electronic medical records. The study was reviewed and approval from the Institutional Review Board of Beijing Chao-yang Hospital, Capital Medical University was obtained.

### Study population and case selection

The cases included in the present study comprised inpatients admitted to our hospital from January 1, 2007, to December 31, 2016. The microbial database revealed 80 cases of patients with positive results of *Salmonella* infection according to the bacterial culture. The clinical information of these 80 patients was screened for selection of adults aged over 18 years who were diagnosed with mycotic aneurysm caused by *Salmonella*. The observation endpoints of these cases included treatment failure, death or loss of follow-up.

In addition, appropriate keywords were selected. The literature with respect to cases of mycotic aneurysm caused by *Salmonella* published on the Chinese Wanfang database and PubMed in the past 10 years was searched, and full-text articles were downloaded. The articles were assessed, and repeated or unqualified cases were removed. The demographic characteristics, clinical manifestations, laboratory and imaging examination, drug administration and surgical approaches, and follow-up data were extracted for analysis.

### Definition

Presently, the diagnostic criteria for *Salmonella*-induced aneurysms include: symptoms of infection, such as fever, chills, chest pain, and back pain; computed tomography imaging showing positive performance; inflammation and surrounding abscess formation observed in surgery; positive results for *Salmonella* or blood culture from aneurysm wall.

Follow-up of patients showed no recurrence. Failure of treatment included recurrence of *Salmonella* bacteremia or mycotic aneurysm caused by other microorganisms during follow-up, or ruptured aneurysms after treatment among other complications, and death.

## Results

### Patient cohort

A total of 4 patients in our hospital were enrolled in the present study. The average age at diagnosis was 61.25 years. All patients were male; two were diagnosed in 2014, and the other two in 2015 and 2016.

The relevant literature of the last 10 years was retrieved, including 10 Chinese and 26 English studies. The Chinese studies reported 17 cases of *Salmonella* infection that caused an aneurysm, whereas the English literature reported 36 cases. The average age of these 53 cases was 65.13 years, 79.2% (42/53) was male. The cases reported in the literature are summarized in Supplementary Table S1 (refs. 22–56). The clinical features of the 4 cases treated at our hospital and the 53 cases reported in the literature are summarized in Table 1.

**Table 1.** Clinical characteristics of *Salmonella*-induced mycotic aneurysms.

	Cases at our center	Cases reported in the literature
Number of patients	4	53
Gender (male/female)	4:1	3.8:1
Mean age (years)	61.25	65.13
Risk factors		
Hypertension	2/4	30/53
Diabetes mellitus	1/4	18/53
Atherosclerosis	1/4	6/53
Hyperlipidemia		2/53
AIDS		2/53
Other factors		CKD, tobacco dependence (2 cases each), malignancy, drug dependence, biological therapies (1 case each)
Signs and symptoms		
Fever	4/4	43/53
Diarrhea (sporadic)	1/4	9/53
Area of aneurysms pain	4/4	25/53
Organisms		
*S. enteritidis*	1/4	17/53
*S. choleraesuis*		13/53
*S. typhi*	1/4	1/53
*S. typhimurium*		3/53
*S. paratyphi B*		2/53
*S. dublin*	2/4	2/53
*S. newport*		1/53
*S. gallinarum*		1/53
Others		13/53 (no specific identification results)
Location of aneurysm		
Abdominal aorta	1/4	36/53
Thoracic aorta and aorta arch	2/4	6/53
Iliac and beyond artery	1/4	6/53
Secondary arteries of aorta		4/53
Coronary artery		1/53
Diameter of aneurysm (cm)	3.0 (2.2,4.7)	4.5 (0.9,14.0)
Complications		
Aneurysm rupture	1/4	9/53
Psoas abscess	1/4	5/53
Surrounding abscess		2/53
Spondylodiscitis		1/53

AIDS: acquired immune deficiency syndrome; CKD: chronic kidney disease.

Among the 57 cases of patients with mycotic aneurysms caused by *Salmonella*, most also presented other complications such as hypertension (32/57), diabetes (19/57), atherosclerosis (7/57), hyperlipidemia (2/57), acquired immunodeficiency syndrome (2/57), chronic kidney disease (2/57), smoking, drug abuse, and autoimmune diseases treated by biological agents.

The majority of patients showed different courses of recurrent fever (47/57), with a moderate-to-high degree in most of them. In addition, most patients presented chills, and some cases presented diarrhea from 2 days to 1 month before the aneurysm was diagnosed (10/53), pain at the site of the aneurysm (29/57), and shock caused by the rupture. Laboratory examination found a significantly increased number of white blood cells (26/29), especially neutrophils, accompanied by the increase of inflammatory markers such as C-reactive protein and dynamic erythrocyte sedimentation rate. Nearly all patients showed a positive blood culture result for *Salmonella* at least once. The results of postoperative tissue culture in some patients were in agreement with those of the blood culture (10/57). The majority of the imaging examinations used for these cases were enhancement computed tomography, vascular CTA, and vascular angiography. Frequently, the aneurysms were found in the abdominal aorta (37/57), primarily below the level of the renal artery, followed by the thoracic aorta and aortic arch (8/57), iliac artery and more distal artery (7/57), secondary artery branched from aorta (4/57), and coronary artery (1/57).

**Figure 1.**
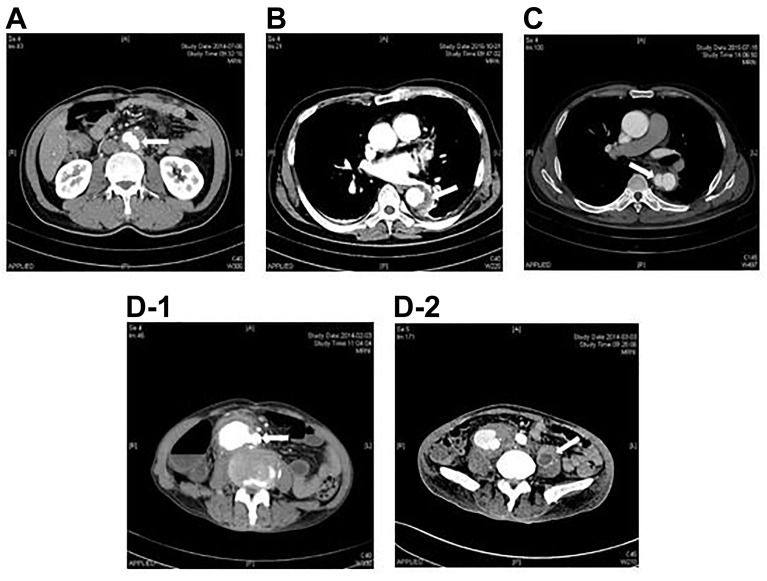
Preoperative enhancement computed tomography of the 4 patients with mycotic aneurysm (lesions: white arrows). *A*, Male, 41-year-old, CT scan of abdomen demonstrating an eccentric dilatation of the blood vessel lumen at the bifurcation of the abdominal aorta and iliac vessels. A filling defect was observed. *B*, Male, 62-year-old, CT scan of chest demonstrating an eccentric thickening of the distal segment of the thoracic aorta accompanied by calcified intimal displacement. *C*, Male, 75-year-old, CT scan of chest demonstrating a saccular aneurysm formation at the thoracic segment of descending aorta and mural filling defect. *D-1,* Male, 67-year-old, CT scan of abdomen showing pseudoaneurysm formation at the abdominal aortic bifurcation and the initial segment of the right iliac artery. *D-2*, Same patient, showing the formation of the left psoas abscess.

The mycotic aneurysm can enlarge rapidly, from a diameter of 0.9 to 14 cm. Among the reported cases, the most common complications included rupture of the aneurysm (10/57), psoas abscess (6/57), aneurysmal abscess (2/57), and discitis (1/57). The imaging findings of the 4 patients in our hospital are shown in Figure 1.

The most common strains reported in the 57 cases were *Salmonella enteritidis* (18/57), followed by *Salmonella choleraesuis* (13/57), *Salmonella typhi* (2/57), *Salmonella dublin* (4/57), *Salmonella typhimurium* (3/57), *Salmonella paratyphi* B (2/57), *Salmonella newport* (1/57), and *Salmonella gallinarum* (1/57). However, 13 patients were not subjected to further identification or the reports did not show the detailed identification results.

### Management and outcome

The treatment of the 57 patients included in this study is described in Table 2. The literature reported the prognosis in 46 cases, while it was not mentioned in 7 cases.

**Table 2.** Treatment options and outcomes of *Salmonella*-induced mycotic aneurysms.

	Cases at our center	Cases reported in the literature
Number of patients	4	53
Antimicrobial		
Quinolones	1/4	13/53
Third-generation cephalosporins	2/4	9/53
Carbapenems		7/53
β-lactam antibiotics	1/4	5/53
Fourth-generation cephalosporins		3/53
Macrolides		1/53
Course of antibiotics (postoperative)	4–6 weeks*	2–24 weeks*
Medical therapy		5/53
Surgical therapy		48/53
Resection and ligation		4/53
Resection and bypass		13/53
EVAR	4/4	31/53
Outcome		
Medical therapy	0	5
Survived and no recurrence		
Recurrence of infection		1/4
Deaths		3/4
Open operation	0	17
Survived and no recurrence		11/14
Recurrence of infection		
Deaths		3/14
EVAR	4	31
Survived and no recurrence	3/4	21/28
Recurrence of infection	1/4	5/28
Deaths		2/28

*One patient received the antimicrobial therapy for life. EVAR: endovascular aneurysm repair.

All patients were treated with antibiotics; the most frequently used antibiotics were quinolones, which were administered to 14 cases. In addition, quinolones were used as continuous postoperative anti-infection antibiotics in the majority of patients. However, third generation cephalosporins were selected in 11 cases, carbapenems in 7 cases, β-lactams in 6 cases, fourth generation cephalosporins in 3 cases, and macrolide antibiotic in 1 case for long-term anti-infection treatment after discharge. The specific type of antibiotics was not mentioned in 15 cases of patients. The description of the treatment course in antibiotics administration differed considerably. The sensitive antibiotics were applied preoperatively in the 4 cases at our center including 3 cases that underwent emergency surgery and another case that was subjected to preoperative anti-infection treatment for 8 weeks and postoperative application of antibiotics for more than 4 weeks. Among them, the lifelong application of antibiotic treatment was performed in one case due to the recurrent infection after endovascular aneurysm repair (EVAR) surgery. Preoperative antibiotic treatment was rarely reported in the cases described previously. The duration of postoperative application of antibiotics was from two weeks to six months. The lifelong application of antibiotic treatment was suggested in one case owing to recurrent *Salmonella* bacteremia after EVAR surgery.

For surgical treatment, the approaches included *in situ* reconstructions, extra-anatomic bypass, and endovascular stent repair. The endovascular stent-graft exclusion was performed in 35/57 cases whereas open surgery was performed in 17 cases. Among these, the extra-anatomic bypass grafts after resection of aneurysm selected in 13 cases included artificial, autologous, and allogeneic blood vessels.

Of the 57 patients, 50 were followed up for 2 months to 4 years. Among these, 8 patients died yielding a mortality rate of 16%. No recurrence was observed during the follow-up period of 35 patients. However, fever and *Salmonella* bacteremia reoccurred in 7 cases. A total of 52 patients underwent open surgery or EVAR intervention (46 cases provided follow up results), and 5 patients died after surgery, with the mortality rate of approximately 10.87%. The mortality rates were 21.43 and 7.14% after open surgery or EVAR intervention, respectively. The recurrence rates of infection were 0 and 17.85%, respectively.

## Discussion

Bacterial aneurysm was first described by Osler in 1885 (4). Its diagnosis was based on the imaging findings of the aneurysm and positive results of bacterial culture or the support of arterial wall histological examination. Prior to the widespread use of antibiotics, syphilis, tuberculosis, and untreated endocarditis were the most common causes of mycotic aneurysms. Since the popularization of antibiotics, reports about mycotic aneurysms had decreased gradually. Recently, the most common cause of mycotic aneurysms reported worldwide has been *Staphylococcus aureus*, followed by *Salmonella* (3).

Gastroenteritis is a common clinical manifestation of *Salmonella* infection, which is self-limiting. However, approximately 5% develop bacteremia (5). *Salmonella* has a strong affinity for large blood vessels, and can easily adhere to the damaged vascular wall, playing a decisive role in the pathogenesis of mycotic aneurysms. The current literature reported that the most important pathophysiological mechanism of mycotic aneurysm is necrosis and rupture of the atherosclerotic vascular wall, which causes the adhesion of bacteria (6). In addition, bacterial embolus could also attach to the vascular branches or small nourishing vessels through blood circulation and invade the arterial wall, causing damage to the structure and gradually developing into the aneurysm (7). Accumulating evidence showed that iatrogenic factors, such as surgery or endovascular procedures, might cause vascular endothelial damage, providing an opportunity to the bacteria in blood circulation to strand in these areas and invade the arterial wall (8). In a small number of cases with a true aneurysm, the bacteria arrived through blood circulation, stranded, and caused infection of the aneurysm wall (6).

This study found that middle-aged and elderly people, males, and those with hypertension, diabetes, and atherosclerosis were more susceptible to this disease. The 4 patients from our hospital were male; 1 patient exhibited a clear history of atherosclerosis, and the other 3 patients presented high-risk factors for atherosclerosis, such as advanced age, hypertension, and diabetes but no clear history of aneurysm. The cause of mycotic aneurysm in such individuals was closely related to the presence of atherosclerosis in the vascular wall. Furthermore, 6 cases with a clear history of atherosclerosis, and 31 cases with high-risk factors for atherosclerosis, such as diabetes, hypertension, and hyperlipidemia were described previously. In addition, other cases with factors that might cause vascular endothelial injury, such as drug addiction and use of biological agents, were also observed. Therefore, the adhesion of *Salmonella* on the site of vascular endothelial injury might be the “second damage” to the vascular lesion, eventually resulting in the damage of the vascular wall structure and developing into the aneurysm. In addition, 43/57 cases included in the study were from East Asia, suggesting that this disease has some regional factors.

The current study found that the mycotic aneurysm caused by *Salmonella* infection was clinically represented as the primary manifestations of bloodstream infection, such as repeated fever and chills. Fifty-one percent of the patients (29/57) showed symptoms of pain at the site of infection caused by the increased local vascular tension during aneurysm formation, while some patients presented diarrhea. Laboratory tests suggested that the proportions of white blood cells, neutrophils, and C-reactive protein were increased, and the blood culture suggested *Salmonella* bacteremia. However, due to non-specificity of the early symptoms, misdiagnosis was common. Therefore, for middle-aged and elderly patients with atherosclerosis, detection of *Salmonella* bacteremia, and other high-risk factors, an imaging examination should be performed to diagnose the formation of aneurysm. Moreover, the symptoms of chest, back, and abdominal pain are suggestive of the disease.

The results of this study showed that the majority of mycotic aneurysms caused by *Salmonella* were in the abdominal aorta (37/57), and some patients present diarrhea when infected with *Salmonella*. Therefore, patients simultaneously presenting diarrhea and abdominal pain are likely to be missed.

The complications of mycotic aneurysm caused by *Salmonella* were often fatal, including aneurysm rupture caused by the rapid increase of aneurysm volume, hemorrhage, bacterial embolism, and vascular occlusion caused by embolization (9). If the mycotic aneurysm is not treated, the incidence of fatal prognosis is extremely high. Kam et al. (10) reported that more than 53% of *Salmonella*-infected aneurysm ruptured. Hsu et al. (11) reported that the mortality was approximately 16–44%. The present study found that the incidence of aneurysm rupture was 17.54%, which was lower than that in the previous reports. This phenomenon might be attributed to the increased level of medical technique and awareness, and hence, more patients with mycotic aneurysms were diagnosed at an early stage preventing aneurysm rupture by emergency surgery. On the other hand, there might be publication bias.

Of the 4 patients with mycotic aneurysm caused by *Salmonella* reported at our center, 1 patient was complicated with psoas abscess. Of the 53 patients included in the literature review, 5 patients reported this complication (10.53%). The aneurysms in these 6 patients occurred in infrarenal aorta and iliac artery, of which 2 cases ruptured before the psoas abscess was found, which could explain the spread of infection post-rupture, as the psoas muscle tissue is loose and can be easily involved. In addition, the psoas muscle is supplied by the external iliac artery, thereby not excluding the possibility that the psoas muscle infection was caused by hematogenous dissemination. The occurrence of psoas abscess can lead to poor efficacy of antibiotic treatment. While active surgical debridement is essential, antibiotic anti-infection treatment should be appropriately prolonged. Previous studies reported that mycotic aneurysm complicated by psoas abscess is relatively rare, with 4% incidence (12), and the most common pathogen was *Salmonella* (13). However, the incidence in this study was much higher than that reported previously. Therefore, patients with repeated fever and other symptoms of poor inflammation control during anti-infection treatment after surgical intervention of mycotic aneurysm should be considered having psoas abscess.

Treatment of patients with mycotic aneurysm caused by *Salmonella* should include antibiotic therapy and surgery. However, preoperative anti-infective treatment is still controversial. Except in patients requiring emergency surgeries, sufficient anti-infection therapy is recommended for reducing the intraoperative risks and postoperative recurrence of infection. The preoperative anti-infection treatment ranged from 1 week to 4 months (11,14). For the postoperative anti-infection treatment, some studies suggested that patients with confirmed or suspected intravascular infection should simultaneously receive surgical treatment and sensitive antibiotic therapy for 6 weeks. In our four patients and the literature, the patients exhibited significant differences in the course of postoperative anti-infection treatment, ranging from 2 weeks to 6 months; 2 cases required lifelong administration of antibiotics. Through the review, 14 cases presented recurrence of infection or death. Among them, 10 cases received surgical intervention, and 6 received postoperative antibiotic treatment for less than 6 weeks, suggesting that the short course of postoperative anti-infection treatment could increase the risk of recurrence. Therefore, in addition to the active treatment with anti-infection antibiotics before surgery, intravenous antibiotics are recommended for at least 6 weeks after surgery (15,16). In addition, close follow-ups are desirable to prevent the recurrence of infection.

Previously, the rate of mortality of patients treated non-surgically was about 70–90% (17). Five patients received non-surgical treatment in this study. Among these, 3 patients died, and 1 patient displayed no prognosis. The rate of mortality was approximately 75%. Because of the high risk of aneurysm rupture and the high mortality of mycotic aneurysms, the surgical treatment should be carried out at the earliest time (7). The review of the literature revealed that the surgical options for mycotic aneurysms included *in situ* reconstruction, extra-anatomic bypass, and endovascular stent repair. EVAR was routine treatment for patients who were not suitable for open surgeries. Because of the rapidly increasing diameter of the mycotic aneurysm, coated stent-graft is beneficial to patients unable to tolerate open surgeries (18). However, the risk of infection recurrence is relatively increased, thereby limiting its application. Huang et al. reported that the 30-day mortality of EVAR-treated mycotic aneurysms was 0%, and the 1-year survival was 81.8% (19). Kan et al. (20) reviewed the cases of EVAR-treated mycotic aneurysms and confirmed that the 30-day and 2-year survivals of EVAR-treated mycotic aneurysms were 90 and 82%, respectively. Nevertheless, 23% of the patients presented persistent infection after surgery, and the 1-year survival of these patients was about 39%. Furthermore, the potential factors of persistent infection after EVAR include fever during operation and aneurysm rupture. In this study, the rate of mortality in EVAR-treated patients was significantly lower than that in open surgery-treated patients (7.14 *vs* 21.43%). During the follow-up period from 2 months to 4 years, 6 patients experienced recurrence of infection after EVAR, which was significantly higher than open surgery-treated patients. Among them, 2 cases reported ruptured aneurysm before surgery and 2 cases stopped the use of antibiotics after surgery, which might be attributed to the postoperative recurrence of infection. Additional reports were also presented for patients indicated for open surgery; a coated stent was first implanted to prevent aneurysm rupture. Subsequent to the stabilization of the general condition and several weeks of sufficient anti-infection treatment, surgery was carried out (21). However, relevant data to prove that the risk of death can be reduced is yet absent. Both surgical methods possess advantages and disadvantages, and hence, additional experimental evidence is imperative to aid clinicians and provide guidance for the choices of surgical methods and operation timing.

In summary, the mortality of mycotic aneurysm caused by *Salmonella* infection was high, and middle-aged and elderly males, hypertension, diabetes, and atherosclerosis were considered risk factors for this disease. The most common infection site of mycotic aneurysm caused by *Salmonella* was the abdominal aorta. Most clinical cases experienced chills and fever, and 51% presented pain and discomfort at the sites of the aneurysm. The most common complication of mycotic aneurysm caused by *Salmonella* was aneurysm rupture. The complication of psoas abscess should be considered if patients with infectious infrarenal aorta and iliac artery aneurysms present persistent signs of infection after surgery. The treatment of patients with a mycotic aneurysm caused by *Salmonella* included antibiotic therapy and surgical treatment. Open surgery was superior to EVAR treatment in clearing the infected foci and reduction the postoperative recurrence. To prevent postoperative recurrence, the recommended intravenous antibiotic therapy should be prolonged until 6 weeks after surgery, especially in patients with EVAR who should also be closely followed up.

## Supplementary Material

Click here to view [pdf]
